# A novel multi-ingredient supplement significantly improves ocular symptom severity and tear production in patients with dry eye disease: results from a randomized, placebo-controlled clinical trial

**DOI:** 10.3389/fopht.2024.1362113

**Published:** 2024-04-24

**Authors:** Neda Gioia, Jeffry Gerson, Robert Ryan, Krista Barbour, Julie Poteet, Brooke Jennings, Matthew Sharp, Ryan Lowery, Jacob Wilson, Abhijeet Morde, Deshanie Rai, Muralidhara Padigaru, Laura M. Periman

**Affiliations:** ^1^ Integrative Vision Corp, Shrewsbury, NJ, United States; ^2^ Grin Eye Care, Olathe, KS, United States; ^3^ Medical Affairs Bausch + Lomb, Bridgewater, NJ, United States; ^4^ MyEyeDr, Acworth, GA, United States; ^5^ Applied Science and Performance Institute, Tampa, FL, United States; ^6^ OmniActive Health Technologies, Mumbai, India; ^7^ Dry Eye Master, Seattle, WA, United States

**Keywords:** dry eye disease, anti-inflammatory nutraceutical, supplements, tear volume, tear quality, lutein, zeaxanthin, curcumin

## Abstract

**Introduction:**

Dry eye disease (DED) is multifactorial and characterized by a loss of tear film homeostasis that causes a cycle of tear film instability, tear hyperosmolarity, and inflammation. While artificial tears are the traditional mainstay of treatment, addressing the underlying pathophysiology could relieve symptoms and prevent progression. Increasing evidence indicates a role for oral nutritional supplementation in multiple ophthalmic diseases, including DED. Lutein, zeaxanthin, curcumin, and vitamin D3 have demonstrated protective and anti-inflammatory properties in ocular models. This prospective, randomized, double-blind, parallel, placebo-controlled study evaluated the efficacy and safety of a proprietary blend of lutein, zeaxanthin isomers, curcumin, and vitamin D3 (LCD) as a daily supplement in adult participants with DED.

**Methods:**

Participants were randomized to receive one LCD supplement capsule (lutein 20 mg, zeaxanthin isomers 4 mg, curcumin 200 mg curcuminoids, and vitamin D3 600 IU) or placebo per day for 8 weeks (LCD, n=77; placebo, n=78). Primary outcomes were changes in tear volume (Schirmer’s test) and ocular symptoms (Ocular Surface Disease Index [OSDI]).

**Results:**

The study met its primary endpoints: the LCD group demonstrated significantly better Schirmer’s test scores and improvement in overall OSDI score, versus placebo, at Day 56 (p<0.001 for both). Scores for total OSDI, and symptoms and vision domains, significantly improved by Day 14 for LCD versus placebo, (p<0.05 for all) and were maintained to Day 56 (p<0.001). In addition, the LCD group demonstrated significantly improved tear film break-up time (TBUT) and tear film osmolarity, versus placebo, by Day 56 (p<0.001), along with significant improvements in corneal and conjunctival staining (p<0.001 for both), and inflammation (matrix metalloproteinase-9; p<0.001 for each eye). Total Standard Patient Evaluation of Eye Dryness (SPEED) score, and scores for the frequency and severity domains, were significantly improved by Day 14 for LCD versus placebo (p<0.05 for all) and maintained to Day 56 (p<0.001). There was no difference between groups for artificial tear usage. The supplement was well-tolerated.

**Discussion:**

Once-daily LCD supplementation significantly improved tear production, stability and quality, reduced ocular surface damage and inflammation, and improved participants’ symptoms. LCD supplementation could offer a useful adjunct to artificial tears for patients with DED (NCT05481450).

## Highlights

- Dry eye disease (DED) affects millions of people. Addressing the self-perpetuating pathophysiology could relieve the chronic discomfort and visual disturbance that significantly impact patients’ activities and wellbeing, and prevent progression caused by accumulating ocular inflammation and damage.
- Increasing evidence indicates a role for oral nutritional supplementation on moderating DED pathophysiology through the impact of bioactive compounds. Specifically, lutein, zeaxanthin, curcumin, and vitamin D3 have protective and anti-inflammatory properties in ocular models and supplementation with a unique blend of these compounds (LCD) improves DED symptoms and alters the inflammatory profile in *in vivo* and in-human studies. This prospective, randomized, double-blind, parallel, placebo-controlled, study evaluated 8-week efficacy and safety of once-daily LCD supplementation in adult participants with DED.
- The study met its primary endpoints and LCD was well-tolerated. This trial highlights the benefits of nutritional support with this proprietary supplement, by improving patients’ experience of DED symptoms and addressing the loss of tear film homeostasis and ocular inflammation that causes them.

## 1 Introduction

Dry eye disease (DED) is a multifactorial disorder characterized by a loss of tear film homeostasis that leads to a self-perpetuating cycle of tear film instability, tear hyperosmolarity, and inflammatory events, resulting in ocular surface inflammation and injury (1–4). The estimated global prevalence of DED ranges from 5 to 50% and risk typically increases with age and female gender, with an increasing prevalence in younger people and schoolchildren (1, 2, 5, 6).

Loss of tear film homeostasis in DED is secondary to ocular dysfunction and caused by one or more contributing factors, including pre-existing eye conditions and systemic diseases, ongoing medication, environmental conditions, and lifestyle choices, including tobacco and electronic device use (1, 2, 6). The resulting increase in tear film evaporation, and/or reduction in tear production, leads to tear deficiency, tear hyperosmolarity, and tear film instability, triggering inflammatory responses (2, 3, 6). The presence of inflammation in patients with DED is associated with increased symptomology, ocular surface irritation, worsening tear dysfunction, and disrupted function of ocular components, including the meibomian glands (7). There is increasing understanding of the driving role inflammation plays in ocular surface changes and the importance of avoiding chronic inflammation to prevent scarring and damage of the ocular surface (7). Matrix metalloproteinase-9 (MMP-9) is an inflammatory mediator that is consistently elevated in the tears of patients with DED and correlates with disease severity (3). DED reduces patients’ overall quality of life and can negatively affect perception of physical and mental wellbeing; recent studies also suggest an association between DED and anxiety and depression. The impact of chronic symptoms on daily activities is also associated with considerable economic impact related to loss of productivity (2, 5).

The goals of DED treatment are to restore and maintain ocular surface homeostasis, minimize symptoms and ocular surface damage, and maximize visual function and patient quality of life (2, 8). Treatment is typically long-term, reflecting the chronic nature of DED, and often integrates pharmacologic and nonpharmacologic approaches as part of a progressive strategy (2, 8). Tear replacement with over-the-counter ocular lubricants aims to replace or enhance the tear film and is considered the mainstay of DED treatment (2, 8).

Lifestyle and environmental changes are also recommended as first steps to address DED symptoms and reduce their impact, and include improving general wellness through adequate sleep, hydration, and nutrition (2, 8). There is increasing interest in oral nutritional supplementation for the management of several ophthalmic diseases, with growing evidence supporting this approach for DED; this is based on the anti-inflammatory and antioxidant effects of bioactive substances from food on DED pathophysiology and their potential for administration as nutraceuticals (8–10). Bioactive compounds that have properties with ocular relevance include omega-3 fatty acids, phytochemicals such as carotenoids and polyphenols, vitamins, and which have demonstrated anti-inflammatory and/or antioxidant activities that affect human metabolic, physiologic, or immunologic processes, although precise dosage, formulation and duration of supplementation are yet to be established (7, 9–11).

Omega-3 fatty acids have established roles in physiological function, including anti-inflammatory, neurological, and metabolic properties, and are an example of a substance with bioactivity that could be relevant to ocular health. Although well-studied, the precise role and benefit of omega-3 fatty acid supplementation in DED remains unclear and continues to be explored through clinical trials and observational studies (7–10, 12).

Lutein and zeaxanthin are carotenoid pigments uniquely concentrated in the human macula and widely recommended as dietary supplements for preventing vision loss from age-related macular degeneration (AMD) (11). Following ingestion as a supplement, lutein has demonstrated antioxidant and anti-inflammatory effects, protecting the retina against photo-oxidative damage and inflammatory cytokine production caused by exposure to blue light (13).

Curcumin is a polyphenol extracted from turmeric that has established anti-inflammatory properties, with evidence demonstrating its effect on oxidative stress and cytokine pathways implicated in the pathogenesis of ophthalmic conditions such as glaucoma, DED, and AMD (10, 14). *In vitro*, curcumin can reduce proinflammatory cytokines in corneal epithelial cells and act as a neuroprotector of retina precursor cells. When used as a supplement in patients with wet AMD, alongside anti-vascular endothelial growth factor injections, curcumin improved visual acuity and reduced the total number of injections patients needed (10, 14). Studies are ongoing to develop formulations to improve topical administration of curcumin to the ocular surface and harness the neuroprotective capabilities in the context of glaucoma and other ophthalmological diseases (10, 14).

Vitamin D3 is a prohormone, with antioxidant, immunomodulatory, and anti-inflammatory properties, which can affect the functions of corneal epithelial cells, including barrier provision and response to inflammation and infection (15). *In vitro*, vitamin D3 exerts anti-inflammatory effects on the cornea by inhibiting stress-induced cellular inflammation or modifying signaling to reduce secretion of proinflammatory cytokines. In patients with DED, low levels of vitamin D3 are associated with increased DED severity, poor tear film stability, and reduced tear volume. Supplementation has been shown to improve the efficacy of artificial tears and reduce disease severity, in both vitamin D3-deficient and non-deficient patients (10, 15–17).

Recent data in patients with DED from India demonstrated that a novel multi-ingredient supplement formulation containing a proprietary blend of lutein, zeaxanthin isomers, curcumin, and vitamin D3 (LCD) significantly improved tear production, stability, and quality, and reduced inflammation and ocular surface damage in patients with mild-to-moderate DED (18). The impact of LCD supplementation had been previously demonstrated in an *in vivo* model of DED in rats, in which the formulation improved tear production and tear film stability, reduced oxidative stress and inflammatory markers, and increased production of tear proteins (19).

The objective of the current study was to evaluate the efficacy of the LCD supplement on tear production and quality, ocular surface symptom severity, and patient experience of disease in a larger population of adult participants with DED.

## 2 Materials and methods

### 2.1 Study design and population

This was a prospective, randomized, double-blind, parallel, placebo-controlled, clinical interventional study to evaluate the efficacy and safety of the LCD supplement in adult participants (aged 18–65) with clinically diagnosed DED. The study was conducted in accordance with the International Conference on Harmonization Good Clinical Practice guidelines (ICH E6-R2) and applicable local regulatory requirements and laws.

Participants were enrolled from four study centers in Tampa Bay, Florida, United States, through word of mouth, email contact, and a recruitment database. Participants were selected based on the eligibility criteria summarized in **Supplementary Table S1**, including age, confirmed DED diagnosis, and symptom assessment, and consideration of relevant medical and non-medical conditions to optimize participant safety and prevent confounding. During recruitment, all participants were screened using the Ocular Surface Disease Index (OSDI) questionnaire and were offered the opportunity to be evaluated for the trial if they were within the inclusionary range (score of 12–40; **Supplementary Table S1**).

For each participant, the total study duration was a maximum of 66 days. This included a screening period of 7 days, a treatment period of 56 days (8 weeks), and an end of study visit at 56 + 3 days; a full medical history and physical examination were performed at the screening visit (**Supplementary Figure S1**). After screening, online software (randomizer.org) was used to randomly assign eligible, consenting participant to either the LCD supplement or placebo arms in a 1:1 ratio (randomization visit, Day 1). Follow-up visits occurred at Days 14, 28, and 56 (**Supplementary Figure S1**).

### 2.2 Study intervention and procedures

Participants in the LCD supplement arm received a 670 mg soft gel capsule containing micronized curcumin extract providing 200 mg total curcuminoids, micronized marigold extract concentrate providing 20 mg lutein and 4 mg zeaxanthin isomers, and 1.5 mg of vitamin D3, providing 600 IU vitamin D3; micronization of curcumin and marigold extracts improves bioavailability through enhanced surface area. Participants in the placebo arm received a 670 mg soft gel capsule containing only soybean oil. Each capsule weighed ~670 mg and was manufactured by OmniActive Health Technologies; full details of LCD supplement production have been previously described (18, 19), and the supplement and placebo formulations are described in **Supplementary Table S2**. Participants were asked to report use of artificial tears as rescue medication in cases where they were unable to tolerate their DED symptoms.

Participants consumed one capsule each morning, at the same time and after food, for 56 days. All enrolled and dosed participants received a diary to record the date and time of capsule consumption, any use of artificial tears, adverse events (AEs), and concomitant medication. Participant diaries were checked at each follow-up visit to assess compliance, which was reported as the mean (standard deviation) percentage of compliant participants in each treatment arm.

### 2.3 Study objectives and assessments

The two primary endpoints were change in Schirmer’s test assessment of tear volume and change in OSDI score, from baseline to Day 56. Secondary efficacy endpoints included changes from baseline to Day 56 in tear film break-up time (TBUT), Standard Patient Evaluation of Eye Dryness (SPEED), corneal and conjunctival staining, tear osmolarity, presence of MMP-9, and information on artificial tear use. Statistical comparisons for study outcomes were made for LCD supplement versus placebo.

Safety endpoints included laboratory assessments (hematology and biochemistry analyses and complete blood count), physical examination (including blood pressure, pulse rate, oxygen saturation, and body temperature), and AE monitoring, based on the Common Terminology Criteria for Adverse Events Version 5.0.

#### 2.3.1 Primary efficacy endpoint evaluation

Primary efficacy endpoints were evaluated at baseline, Day 14, Day 28, and Day 56.

Evaluation of tear volume by length of wetting time of Schirmer’s test strips was performed for each eye, without topical anesthesia, as previously described (20). Briefly, sterile Schirmer’s strips were placed into the lower temporal lid margin of both eyes for 5 minutes and the length of moistened area measured once the strips were removed. The primary tear volume endpoint was mean change in length of wetting time from baseline to Day 56.

Participant perception of the symptomatic and functional effects of DED was evaluated using the change in OSDI questionnaire total score from baseline to Day 56 as the primary endpoint; individual scores for the symptom, vision, and environmental OSDI domains were also available for analysis. Participants used a linear scale to respond to the questions at each visit, based on a linear scoring scale from 0–4. **Supplementary Table S3** provides further details on the OSDI assessment.

#### 2.3.2 Secondary efficacy endpoint evaluation

TBUT and SPEED were evaluated at baseline, Day 14, Day 28, and Day 56; corneal and conjunctival staining, tear osmolarity, and presence of MMP-9 were evaluated at baseline and Day 56.

TBUT was performed using sterile saline to instill the stain-impregnated end of a sterile fluorescein paper strip, which was applied to participants’ eyes and the stain distributed by multiple blinks. Participants were instructed to stop blinking and eyes were immediately examined under a cobalt blue light with a yellow filter using a slit lamp; time from last blink to the first appearance of a distinct break-up in tear film was recorded, in seconds, for both eyes. All four centers were familiar with TBUT as part of their routine clinical practice and this assessment was chosen over non-invasive tear break-up time (NITBUT) to ensure familiarity with the protocol.

Following the application of fluorescein for TBUT assessment, both eyes were examined for corneal and conjunctival staining and severity of ocular surface damage was graded using the Efron scale, based on coloration (**Supplementary Table S3**).

The SPEED questionnaire was used to quantify participant perception of the symptomatic and functional effects of DED and completed by participants to give a total score for each visit and scores for the severity and frequency domains. **Supplementary Table S2** provides further details on the assessment of TBUT, SPEED, and corneal and conjunctival staining.

Tear osmolarity was evaluated for each eye using the TearLab Osmolarity System (TearLab Corp., San Diego, California, USA), according to instructions, using samples collected from the temporal side of the lower tear meniscus without pulling the lower eyelid. Presence of MMP-9 was qualitatively determined for each eye using InflammaDry^®^ MMP-9 test kits (QUIDEL^®^, San Diego, CA, USA): positive tests indicated presence of MMP-9 at a concentration of ≥40 ng/mL and a negative result indicated presence of MMP-9 at a concentration <40 ng/mL.

Artificial tear use was assessed at all study visits; the number of participants using artificial tears, and the frequency of use, was reported for each group at each study time point.

#### 2.3.3 Adverse event reporting

Participants were asked to report AEs as soon as possible by email and/or at each visit, including incidences of bloating, diarrhea, heartburn, nausea, constipation, upset stomach, headache, abdominal discomfort, or any other events they did not consider normal. The study would be stopped if any severe AEs were reported, such as a life-threatening event, hospitalization, disability, or permanent injury. AE frequency was defined as how many participants reported no AE (0), reported an AE once during the study (1), or reported an AE twice during the study (2). Severity was defined as no AE (0), mild AE (1), moderately severe AE (2), or severe AE (3).

### 2.4 Statistical analyses

The target randomization number of 124 participants was established to enable at least 110 evaluable participants to complete the study in total (55 in the LCD group and 55 in the placebo group). This number assumed that the test product (LCD) was statistically superior to placebo (two sided, α=0.05, Z-test), with a minimum 80% power and 230% as a standard deviation leading to a mean difference of 73.60% in tear volume according to Schirmer’s Test from Baseline to day 56.

Endpoint analyses were performed between groups and within groups (changes from baseline and each visit). Linear mixed model analysis was used to analyze changes between data collection time points (Days 0, 14, 28, and 56) and differences between groups. In this procedure, time point was a repeated factor and both time point and experimental group (LCD or placebo) were fixed factors. Corneal and conjunctival staining values were reported as whole numbers included in the linear mixed model analyses following assessment by an independent, masked statistician to determine that the skewness and kurtosis values were within acceptable limits to indicate that the data did not deviate from the theoretical normal distribution.

Comparisons of groups across the time points for MMP-9 and artificial tear use (yes–no binary variable) were conducted using Chi square. Pairwise comparisons within linear mixed models were conducted using *post hoc* t-tests, with Bonferroni correction. Separate pairwise comparisons by groups and time points were performed using paired samples t-tests, repeated measures Analysis of Variance, or Mann–Whitney U test, depending on the properties of the data. The Mann–Whitney U test was also used to compare the central tendencies of artificial tear use in the two groups.

## 3 Results

**Figure 1:**
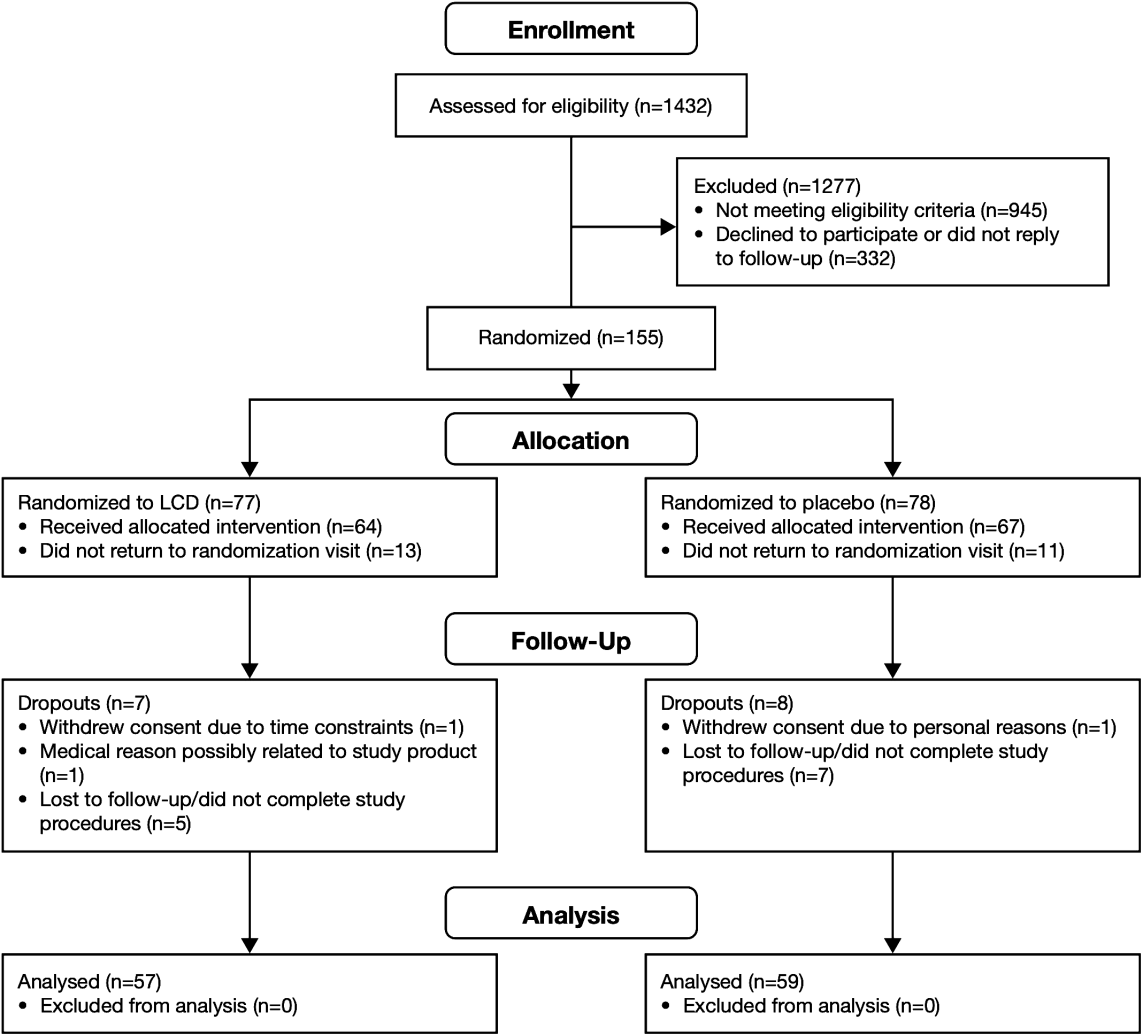
Patient disposition. LCD, lutein, zeaxanthin isomer, curcumin, and vitamin D3.

A total of 1432 participants were screened, of which 1277 were excluded based on ineligibility (n=945; outside of the OSDI inclusionary range) or lack of response to follow-up (n=332). Of the 155 randomized participants, 77 were allocated to receive once-daily LCD supplementation (LCD group) and 78 to once-daily placebo; 64 participants in the LCD group and 67 participants in the placebo group successfully collected their allocated treatment to participate in the study. A total of 116 participants completed the study and were included in the analysis: 57 participants in the LCD group (23 males and 34 females) and 59 participants in the placebo group (28 males and 31 females; **Figure 1**).

Demographic characteristics are shown in **Table 1** and were comparable between the two study groups, including use of corrective eye wear (contact lenses or spectacles). Compliance was high and similar for the LCD and placebo groups (96.89 [3.41] and 96.13 [3.23]% for the LCD group and placebo, respectively).

**Table 1:** Baseline physical characteristics.

	LCD (N=57, m=23, f=34)				Placebo (N=59, m=28, f=31)			
	Mean ± SD	SEM	Median	Min, max	Mean ± SD	SEM	Median	Min, max
**Age (years)**	41.53 ± 11.40	1.51	37	23, 65	42.17 ± 12.07	1.57	41	23, 65
**Height (m)**	1.68 ± 0.10	0.01	1.68	1.47, 1.93	1.73 ± 0.09	0.01	1.72	1.55, 1.91
**Weight (kg)**	75.28 ± 18.95	2.51	73.03	43, 123.6	80.36 ± 13.79	1.8	79.83	52.16, 108.89
**BMI (kg/m^2^)**	26.31 ± 5.15	0.68	25	18.64, 38.58	26.79 ± 4.16	0.52	26.84	18.4, 39.22
**Systolic BP (mmHg)**	123.02 ± 9.50	1.26	123	103, 143	123.07 ± 7.71	1	122	108, 140
**Diastolic BP (mmHg)**	78.16 ± 8.48	1.12	76	62, 98	76.34 ± 5.90	0.77	76	64, 90
**Heart rate (bpm)**	72.86 ± 9.60	1.27	74	50, 93	70.17 ± 9.69	1.26	70	52, 94
**Temperature (°F)**	98.03 ± 0.46	0.06	98	96.8, 98.7	98.00 ± 0.42	0.05	98	96.8, 98.8
**Oxygen saturation (%)**	98.02 ± 0.70	0.09	98	97, 99	97.97 ± 0.77	0.1	98	96, 99
Corrective eyewear								
None Contact lenses Spectacles	8 (14.0%) 21 (36.9%) 28 (49.1%)				9 (15.3%) 24 (40.7%) 26 (44.0%)			

BMI, body mass index; BP, blood pressure; bpm, beats per minute; f, female; LCD, lutein, zeaxanthin isomer, curcumin, and vitamin D3; m, male; SEM, standard error of the mean; SD, standard deviation.

### 3.1 Primary efficacy outcomes

#### 3.1.1 Schirmer’s test

**Figure 2:**
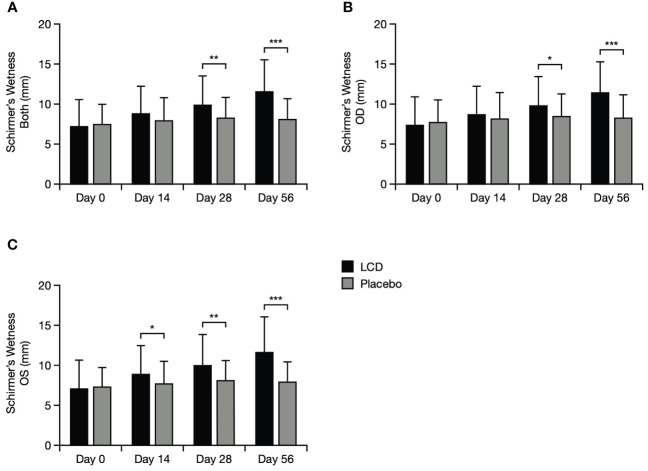
Schirmer’s test results for the mean of both eyes **(A)**, the right eye **(B)**, and the left eye **(C)**. *p<0.05; **p<0.01; ***p<0.001. A value of >10–15 mm is generally considered normal for Schirmer’s test performed without anasthesia (21, 22). Data are presented as mean ± standard deviation; values for the standard error of the mean are presented in **Supplementary Table S5**. LCD, lutein, zeaxanthin isomer, curcumin, and vitamin D3; OD, right eye; OS, left eye.

The LCD group demonstrated significantly better Schirmer’s test results, versus the placebo group, in left and right eyes at Day 28 (p<0.05 for each eye) and Day 56 (p<0.001 for each eye), and additionally for the left eye at Day 14 (p<0.05). The overall mean for both eyes was also significantly better for the LCD group, versus the placebo group, at Day 28 and Day 56 (p<0.05 and p<0.001, respectively; **Figure 2**).

#### 3.1.2 OSDI

**Figure 3:**
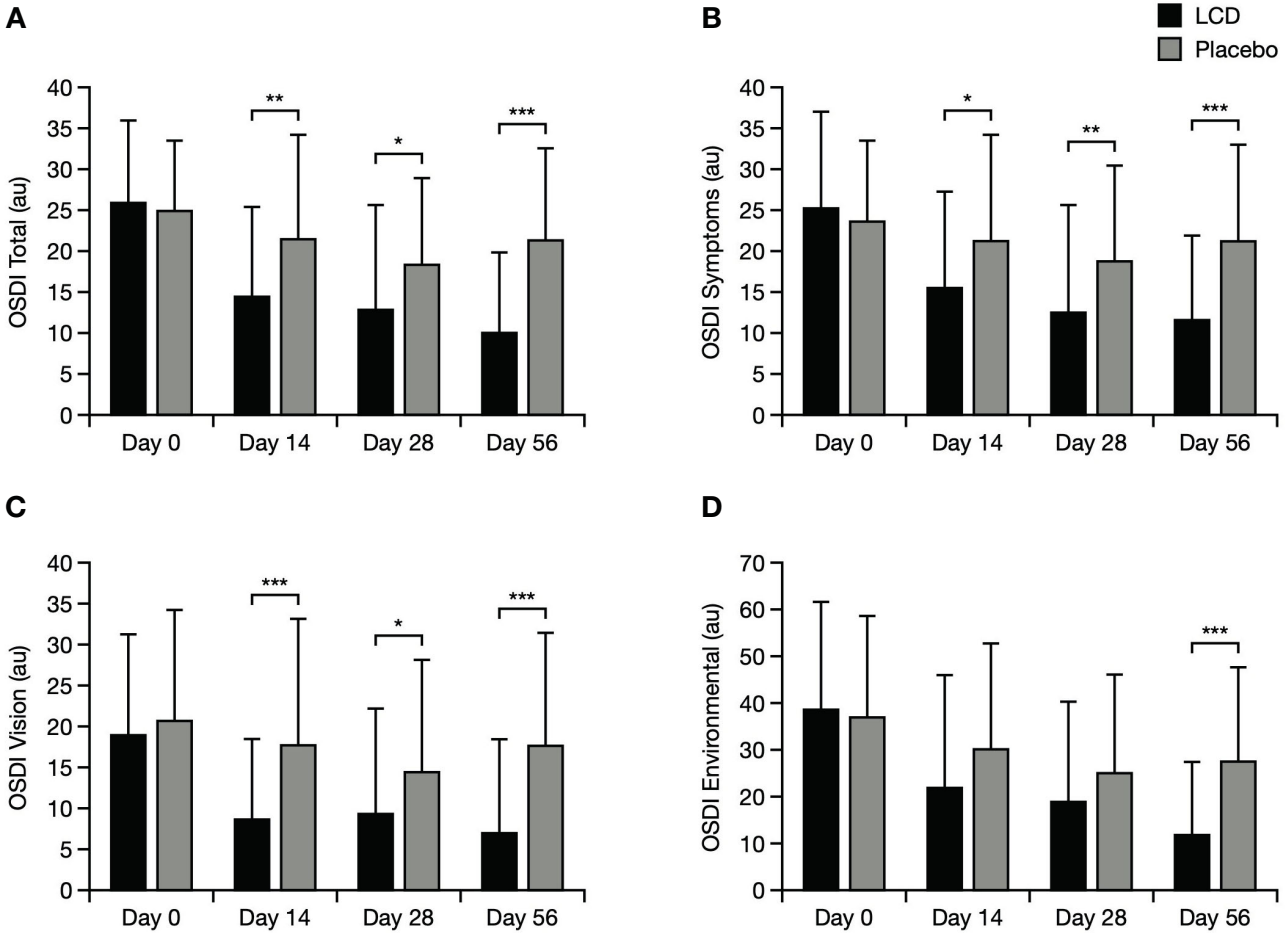
OSDI results for the total score **(A)**, symptoms domain **(B)**, vision domain **(C)**, and environmental domain **(D)**. *p<0.05; **p<0.01; ***p<0.001. The normal range for OSDI score is generally accepted as 0–12 points (23). Data are presented as mean ± standard deviation; values for the standard error of the mean are presented in **Supplementary Table S5**. Au, arbitrary units; LCD, lutein, zeaxanthin isomer, curcumin, and vitamin D3; OSDI, Ocular Surface Disease Index.

Improvement from baseline in total OSDI score was significantly better (lower scores) for participants in the LCD group, versus the placebo group, by Day 14 (p<0.01) and this result was maintained to Days 28 and 56 (p<0.05 and p<0.001, respectively; **Figure 3**). Scores for the OSDI symptoms and vision domains were also significantly better for participants in the LCD group, versus the placebo group, at Day 14 (p<0.05), Day 28 (p<0.05), and Day 56 (p<0.001). The difference in scores for the OSDI environmental domain became significant versus the placebo group at Day 56 (p<0.001).

### 3.2 Secondary efficacy outcomes

#### 3.2.1 TBUT

The LCD group had significant improvement in mean TBUT values, versus the placebo group, in the left eye, right eye, and the mean of both eyes at Day 56 (p<0.001 for each). At Day 28, values for the left eye and the mean of both eyes were also significantly improved, versus the placebo group (p<0.05 for both; **Supplementary Figure S2**).

#### 3.2.2 SPEED

**Figure 4:**
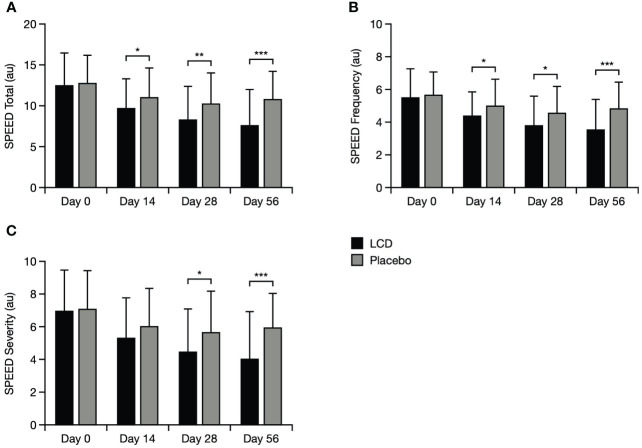
SPEED results for the total score **(A)**, symptoms frequency domain **(B)**, and symptoms severity domain **(C)**. *p<0.05; **p<0.01; ***p<0.001. The SPEED score can range from 0–28 points, with categories for mild, moderate and severe DED defined as 0–4, 5–7 and > 8, respectively (24). Data are presented as mean ± standard deviation; values for the standard error of the mean are presented in **Supplementary Table S5**. Au, arbitrary units; LCD, lutein, zeaxanthin isomer, curcumin, and vitamin D3; SPEED, Standard Patient Evaluation of Eye Dryness.

For participants in the LCD group, improvement from baseline in total SPEED score was significantly better (lower scores) by Day 14, both within the LCD group (p<0.001) and versus the placebo group (p<0.05), and this improvement was maintained to Days 28 and 56 (p<0.001 for each timepoint within the LCD group, and p<0.05 and p<0.001, respectively, versus placebo; **Figure 4**). Scores specifically for the frequency domain mirrored the pattern for total scores and were significantly better for participants in the LCD group at Days 14, 28, and 56, both within the group (p<0.001 for each timepoint) and versus the placebo group (p<0.05, p<0.05, p<0.001, respectively). Scores for the severity domain in the LCD group also decreased from baseline to Day 14, and were significant within the group (p<0.001) at all timepoints; versus placebo, this improvement in severity became significant at Day 28 (p<0.05) and Day 56 (p<0.001).

#### 3.2.3 Corneal and conjunctival staining and tear osmolarity

**Figure 5:**
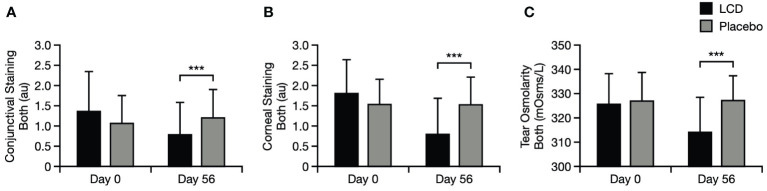
Conjunctival staining test results for the mean of both eyes **(A)**, corneal staining test results for the mean of both eyes **(B)**, and tear osmolarity results for the mean of both eyes **(C)**. ***p<0.001. Literature values for tear osmolarity are in the approximate range of 294–310 mOsm/L (25). Data are presented as mean ± standard deviation; values for the standard error of the mean are presented in **Supplementary Table S5**. au, arbitrary units; LCD, lutein, zeaxanthin isomer, curcumin, and vitamin D3, mOsm/L, milliosmoles per liter.

Corneal staining, conjunctival staining, and tear osmolarity were significantly improved for the LCD group, versus the placebo group, at Day 56. Mean staining values and osmolarity values for both eyes in the LCD group were significantly lower, versus the placebo group, at Day 56 (p<0.001 for all values; **Figure 5**). Staining values and osmolarity values specifically in the right eye and left eye were also significantly lower, versus the placebo group, at Day 56 (p<0.01 for staining values, p<0.001 for osmolarity values, respectively; **Supplementary Figure S3**).

#### 3.2.4 MMP-9

**Figure 6:**
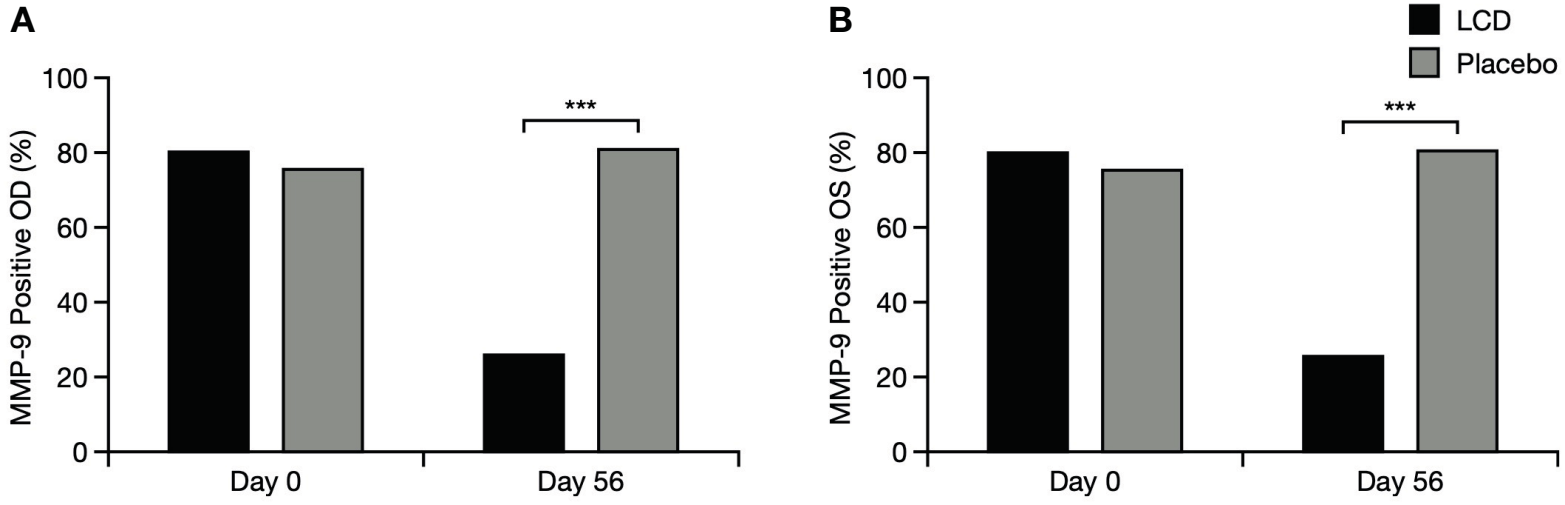
Positive MMP-9 test results for the right eye **(A)** and left eye **(B)**. % indicates percentage of participants who tested positive; ***p<0.001. OD, right eye; OS, left eye; LCD, lutein, zeaxanthin isomer, curcumin, and vitamin D3; MMP-9, matrix metalloproteinase-9.

There was a significant difference in the presence of MMP-9 between the LCD group and the placebo group for the right and left eyes (p<0.001 for each eye): incidence of positive MMP-9 test results decreased from baseline in both eyes for the LCD group (right eye, –67.39%; left eye, –61.36%) but did not decrease for the placebo group (right eye, +6.67%; left eye, +8.70%; **Figure 6**).

#### 3.2.5 Use of artificial tears

Artificial tears used by participants in the study were either carboxymethylcellulose or hydroxypropyl methylcellulose. There was no difference between groups for artificial tear use at any time point, either in terms of reported frequency of use (**Supplementary Table S4**) or the total number of participants using artificial tears. At baseline, 35 participants in each group reported using artificial tears (63.16 and 59.32% for the LCD group and placebo group, respectively); at Day 56, this decreased to 24 and 28 participants for the LCD group and placebo group, respectively (42.11 and 47.46%, respectively; p=0.562; **Supplementary Figure S4**).

### 3.3 Safety

There were no clinically meaningful differences detected in blood safety values or resting vital signs between the LCD and placebo groups.

Two AEs were reported during the study: increased nasal bleeding in one participant in the LCD group and increased blurred vision in one participant in the placebo group (**Table 2**). No drug treatments were used to intervene with either AE reported in this study and no serious AEs occurred during the study.

**Table 2:** Overall summary of AEs.

AEs	LCD (N=57) n (%)	Placebo (N=59) n (%)	Overall (N=116) n (%)
Participants reporting at least one AE	1 (1.75)	1 (1.69)	2 (1.72)
Total number of AEs reported	3 (5.26)	1 (1.69)	4
Total number of SAEs reported	0	0	0
Participants reporting serious AEs	0	0	0
Participants reporting drug-related AEs	0	0	0
Participants reporting AEs leading to early discontinuation	1 (1.75)	0	1 (0.86)
Number of deaths	0	0	0

Data presented as n (%), number of participants and percentage of the sample size.

AE, adverse event; LCD, lutein, zeaxanthin isomer, curcumin, and vitamin D3; SAE, serious adverse event.

The participant who experienced nasal bleeding reported this AE three times (Days 5, 6, and 10) and reported a history of nasal bleeding triggered by vitamin D3 supplementation. After the first two events, LCD supplementation was paused for 2 days (Days 7 and 8) on the advice of the study investigator, during which time no AEs occurred. One day after restarting LCD supplementation, the third nasal bleeding event occurred, and the participant withdrew consent and discontinued the study; this AE was considered mild and possibly related to the study intervention. The participant who reported the incidence of blurred vision remained in the study and completed the study period; this AE was reported once, considered mild in severity and not related to the study intervention.

## 4 Discussion

DED is a chronic disease, with symptoms that cause significant and wide-ranging morbidity (1–4). Along with artificial tears, lifestyle changes are among the first approaches recommended to mitigate the impact of DED symptoms, and there is growing interest in the potential for oral nutritional supplementation, based on the impact of bioactive compounds on elements of DED pathophysiology (2, 8–10). The unique, proprietary blend of lutein, zeaxanthin isomers, curcumin, and vitamin D3 in the LCD supplement has shown therapeutic potential when administered as a once-daily capsule to patients with DED, including ameliorating symptoms and improving tear volume and quantity (18).

In this randomized, placebo-controlled, multicenter study, we explored the efficacy and safety of once-daily LCD supplementation in participants with DED in the United States. Participant compliance in both arms was >96%, and the study met its primary objectives, demonstrating significant improvement in tear volume and ocular symptoms, with the LCD supplement versus placebo, from baseline to Day 56.

Daily consumption of the LCD supplement was associated with significant improvements in tear volume, quality, and tear film stability by Day 56, as measured by change in Schirmer’s test, tear osmolarity, and TBUT. Improvement in these three measures suggests the potential impact of the daily LCD supplement on restoring tear film homeostasis in patients with DED, with implications for breaking the cycle associated with perpetuating chronic symptoms of this disease.

Consuming the LCD supplement daily was also associated with evidence of significant improvements in ocular tissue disruption and inflammation, as assessed by corneal and conjunctival staining, and presence of MMP-9 at Day 56. Taken together with the data on improved tear quantity and quality, these findings suggest an association between the daily LCD supplement and improvement in inflammatory processes that drive the pathophysiology of DED.

Significant improvements in participant-reported ocular symptoms of DED, and participant experience of these symptoms, were associated with daily consumption of the LCD supplement, as assessed by the OSDI and SPEED questionnaires. The scores for both measures significantly improved by 2 weeks and improvements were maintained to Day 56.

The OSDI domains assess DED symptoms, their impact on vision-related daily function in patients’ lives, and patient susceptibility to environmental triggers of DED (26, 27). In this study, daily consumption of the LCD supplement specifically improved DED symptoms and their impact on vision-related function within 2 weeks and maintained this improvement to Day 56; the impact of environmental triggers was also improved by the end of the study. The improvement in both OSDI and TBUT in this study is also consistent with the observation that poor OSDI scores correlate with poor TBUT values (27).

The SPEED questionnaire tracks progression of DED symptoms over time, based on patient experience of sensations of dryness, grittiness, soreness, irritation, burning or watering, and eye fatigue and their bearability (ranging from “not problematic” to “intolerable”) (27, 28). Findings from this study demonstrated significant improvement in participant experience of the frequency and severity of DED symptoms from as early as 2 weeks.

The LCD supplement was well-tolerated, without clinically meaningful changes in blood safety measures or resting vital signs, and participants could continue artificial tear use, if preferred, as indicated by no change in artificial tear use AEs. No serious AEs were reported during the study and the two AEs did not require medical intervention to manage. The event leading to the discontinuation of one participant was considered related to the LCD supplement, possibly resulting from a pre-existing sensitivity to vitamin D3 supplementation.

The findings presented here should be considered in the context of the limitations of the study. The LCD supplement was only administered for 8 weeks, and longer-term follow-up would provide valuable insight into the potential for sustained benefits, which is particularly relevant in the context of the chronic nature of DED. Data from a population of patients with DED in India are broadly similar and confirmatory of the findings presented here, but the benefits of daily consumption of the LCD supplement warrant further study in a larger population of patients with DED. Incorporating NITBUT into future studies as an assessment of tear break-up time, alongside TBUT, could prove informative. Assessment of any impact on ocular lubricant use beyond artificial tears could also be of interest.

## 5 Conclusion

Daily consumption of the LCD supplement significantly improved the production, stability, and quality of tears, with evidence of significant reduction in ocular surface damage and inflammation, and could offer a useful adjunct to artificial tears for patients with DED. Once-daily administration of the LCD supplement was also effective in reducing dry eye symptomology and improving patient experience of DED symptoms, with significant changes reported by 2 weeks in some measures.

This trial highlights the benefits to patients with DED of anti-inflammatory and antioxidant nutritional support with this proprietary supplement, by improving their experience of symptoms, addressing the characteristic loss of tear homeostasis, and ameliorating the ocular inflammation and damage that are the basis of DED pathophysiology.

## Data availability statement

The datasets presented in this article are not readily available because data are held by Bausch + Lomb Inc. Requests to access the datasets should be directed to robert.ryan@bausch.com.

## Ethics statement

The studies involving humans were approved by Advarra Inc., Columbia, Maryland, USA (Pro00063707). The studies were conducted in accordance with the local legislation and institutional requirements. The participants provided their written informed consent to participate in this study.

## Author contributions

NG: Writing – review & editing. JG: Writing – review & editing. RR: Writing – review & editing. KB: Formal Analysis, Visualization, Writing – review & editing. JP: Writing – review & editing. BJ: Formal analysis, Writing – review & editing. MS: Formal analysis, Writing – review & editing. RL: Formal analysis, Writing – review & editing. JW: Formal analysis, Writing – review & editing. AM: Methodology, Formal analysis, Investigation, Writing – review & editing. DR: Formal analysis, Investigation, Writing – review & editing. MP: Methodology, Formal analysis, Investigation, Writing – review & editing. LP: Writing – review & editing.
