# Supplementary figures and images for: A novel multi-ingredient supplement significantly improves ocular symptom severity and tear production in patients with dry eye disease: results from a randomized, placebo-controlled clinical trial
Source: Front Ophthalmol (Lausanne). 2024 Apr 24;4:1362113. doi: 10.3389/fopht.2024.1362113 (PMC11182317; doi:10.3389/fopht.2024.1362113)

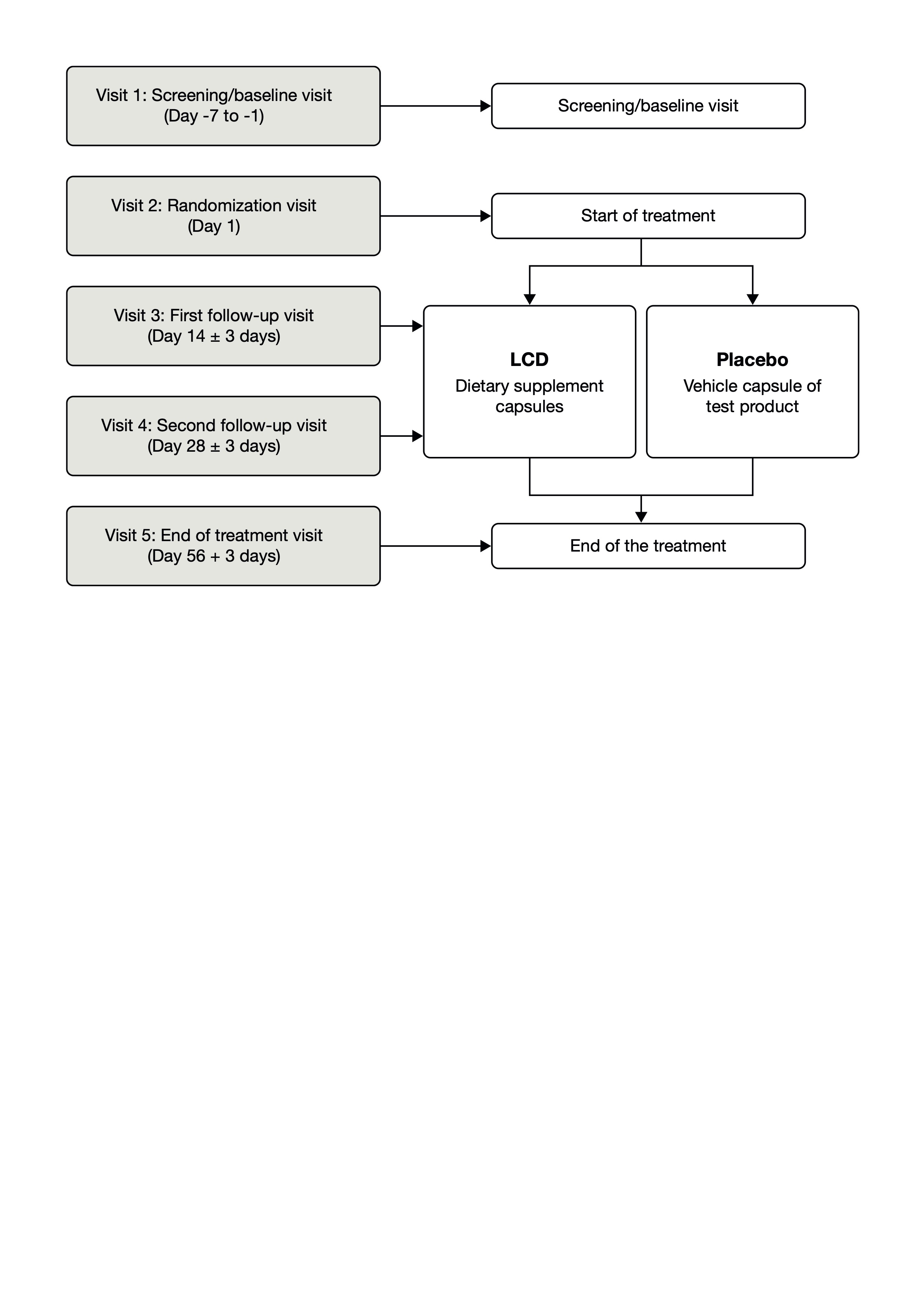

Supplement: Supplementary file 1 [file Image_1.jpeg]

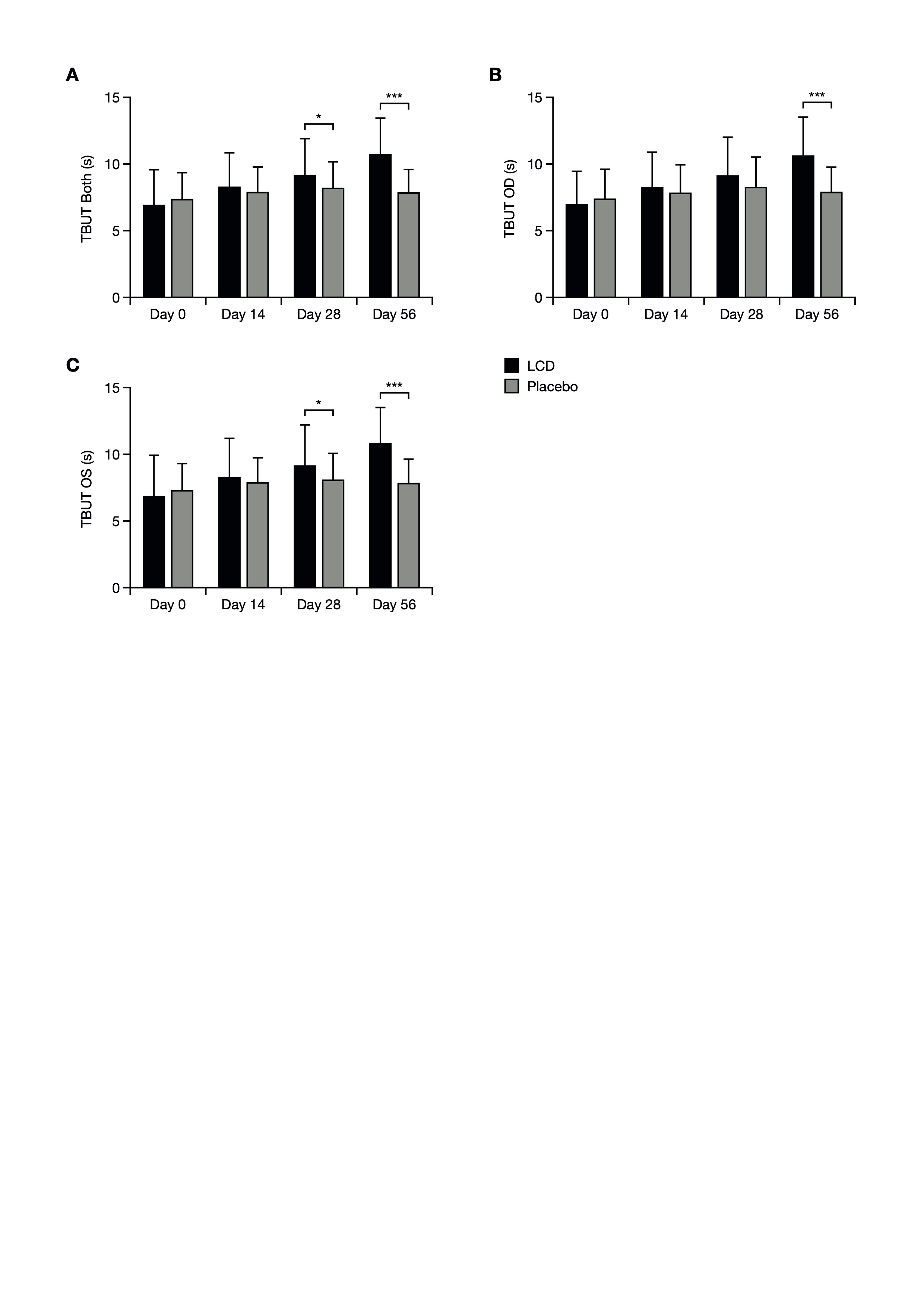

Supplement: Supplementary file 2 [file Image_2.jpeg]

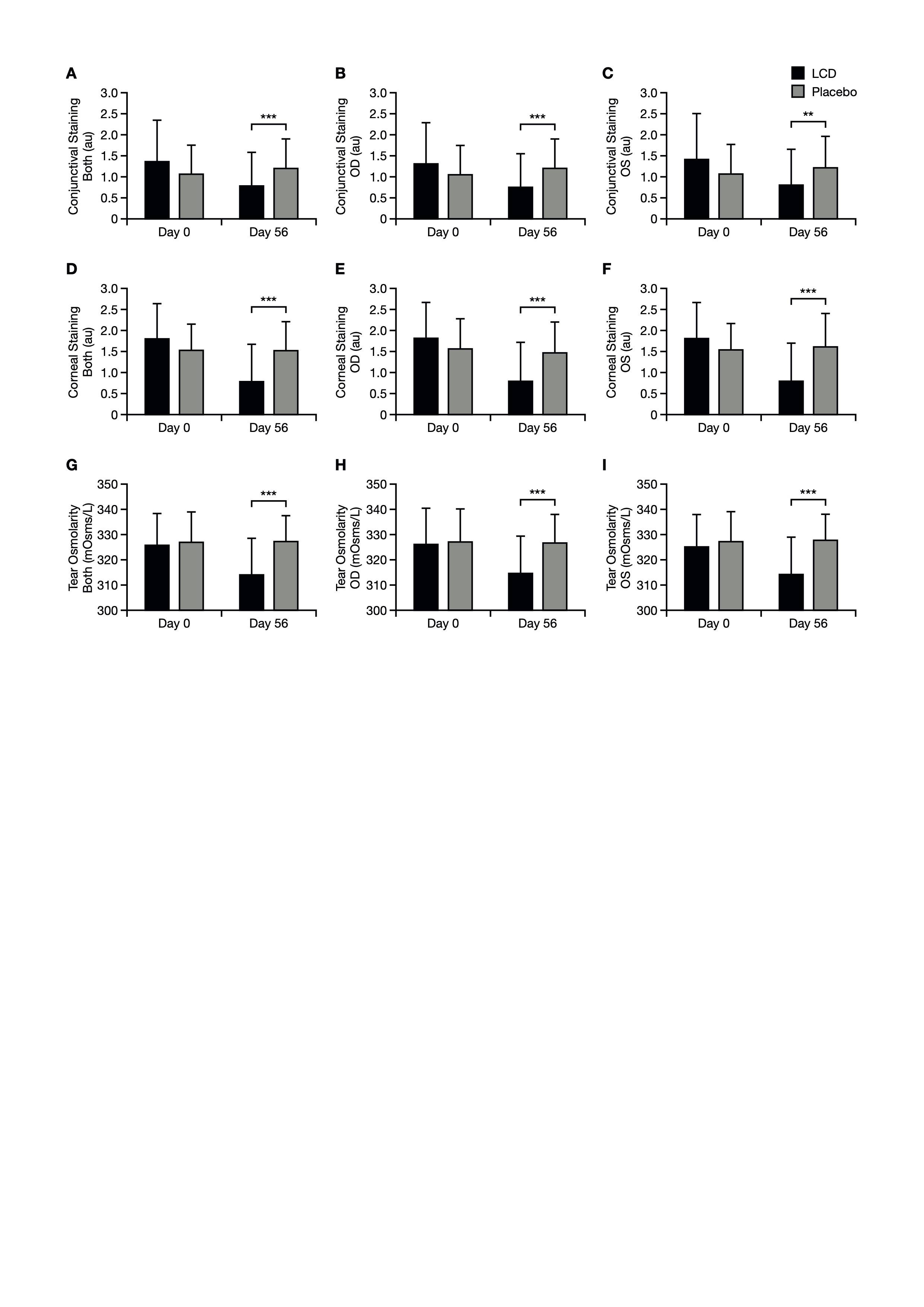

Supplement: Supplementary file 3 [file Image_3.jpeg]

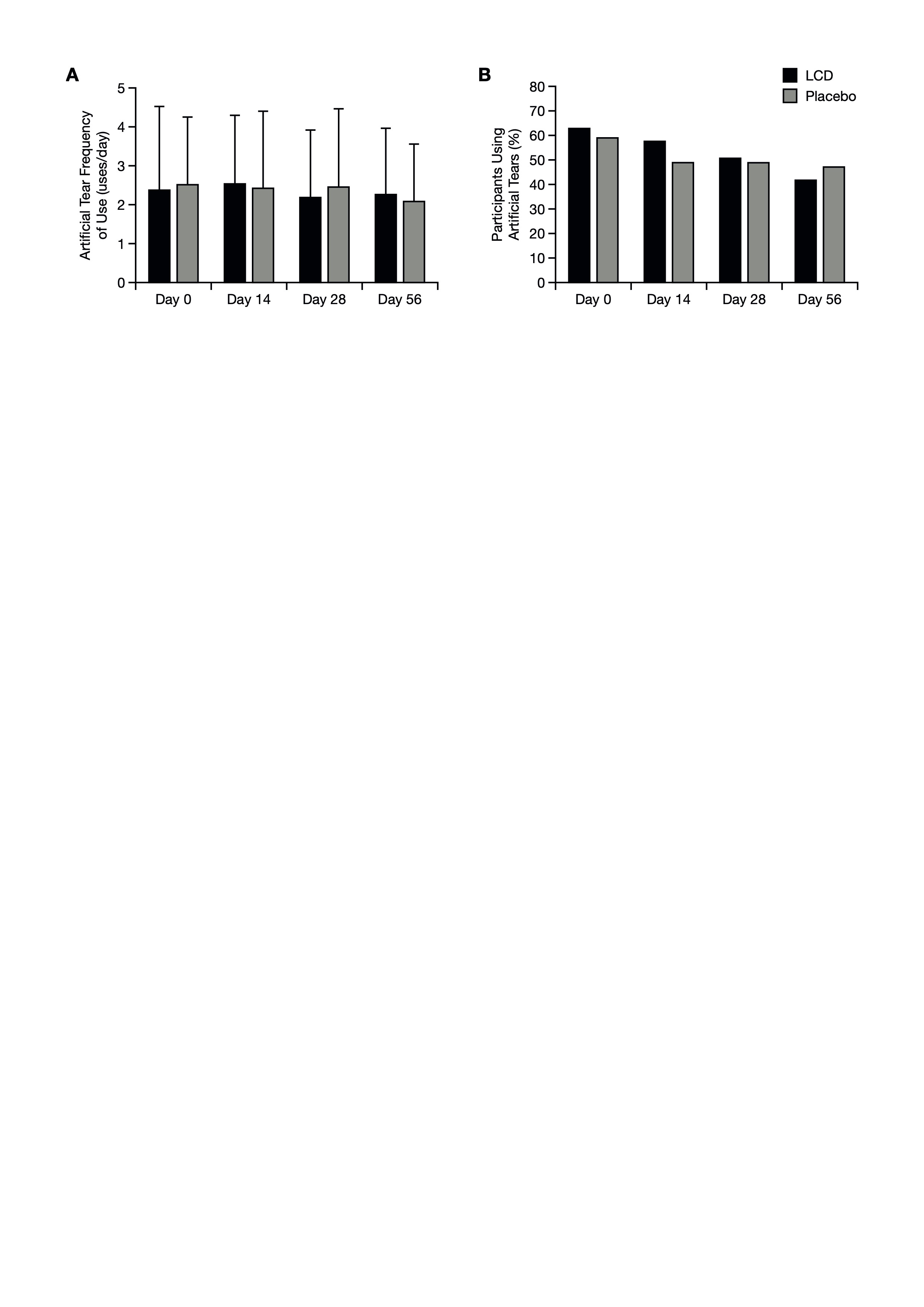

Supplement: Supplementary file 4 [file Image_4.jpeg]
