# Supplementary material for: A novel multi-ingredient supplement significantly improves ocular symptom severity and tear production in patients with dry eye disease: results from a randomized, placebo-controlled clinical trial
Source: Front Ophthalmol (Lausanne). 2024 Apr 24;4:1362113. doi: 10.3389/fopht.2024.1362113 (PMC11182317; doi:10.3389/fopht.2024.1362113)
Supplement: Supplementary file 5 [file DataSheet_1.docx]

**Supplement to:**

**A Novel Multi-Ingredient Supplement Significantly Improves Ocular Symptom Severity and Tear Production in Patients with Dry Eye Disease: Results from a Randomized, Placebo-Controlled Clinical Trial**

**Neda Gioia^1^*, Jeffry Gerson^2^, Robert Ryan^3^, Krista A. Barbour^3^, Julie Poteet^4^, Brooke Jennings^5^, Matthew Sharp^5^, Ryan Lowery^5^, Jacob Wilson^5^, Abhijeet Morde^6^, Deshanie Rai^6^,
Muralidhara Padigaru^6^, Laura M. Periman^7^**

^1^Integrative Vision Corp, Shrewsbury, New Jersey, United States

^2^Grin Eye Care, Olathe, Kansas, United States

^3^Bausch + Lomb, Bridgewater, New Jersey, United States

^4^MyEyeDr, Acworth, Georgia, United States

^5^Applied Science and Performance Institute, Tampa, Florida, United States

^6^OmniActive Health Technologies, Mumbai, India

^7^Dry Eye Master, Seattle, Washington, United States

***Correspondence:**
Neda Gioia
[drdibaee@gmail.com](mailto:drdibaee@gmail.com)

Supplementary Material

**Figure S1.** Study design.

The screening visit included a medical history and physical examination. Baseline information included sex, age, body weight, height, body mass index, medical history, concomitant medication (including oral contraceptive use), digital screen exposure, known allergies and drug reactions, and contact lens and spectacle use. Other baseline clinical characteristics included detailed ocular examination and vital signs (blood pressure, pulse rate, oxygen saturation, and body temperature).

LCD, lutein, zeaxanthin isomer, curcumin, and vitamin D3.

**Figure S2.** Tear film break-up time test results for the mean of both eyes (A), the right eye (B),
and the left eye (C).

*, p<0.05; ***, p<0.001.

A value of > 10 s for TBUT is generally accepted as normal (Vidas Pauk S, Petriček I, Jukić T, Popović-Suić S, Tomić M, Kalauz M, Jandroković S, Masnec S. Noninvasive Tear Film Break-up Time Assessment Using Handheld Lipid Layer Examination Instrument. *Acta Clin Croat* (2019) 58(1):63-71).

Data are presented as mean ± standard deviation; values for the standard error of the mean are presented in **Table S4**.

LCD, lutein, zeaxanthin isomer, curcumin, and vitamin D3; OD, right eye; OS, left eye; s, seconds; TBUT, tear film break-up time.

**Figure S3.** Conjunctival staining results for both eyes (A), the right eye (B), and the left eye (C); corneal staining results for both eyes (D) the right eye (E), and the left eye (F); tear osmolarity results for both eyes (G), the right eye (H), and the left eye (I).

b, *<0.01; ***, p<0.001.

Literature values for tear osmolarity are in the approximate range of 294–310 mOsm/L (Potvin R, Makari S, Rapuano CJ. Tear film osmolarity and dry eye disease: a review of the literature. *Clin Ophthalmol* (2015) 9:2039-47).

Data are presented as mean ± standard deviation; values for the standard error of the mean are presented in **Table S4**.

au, arbitrary units; LCD, lutein, zeaxanthin isomer, curcumin, and vitamin D3; mOsm/L, milliosmoles per liter;

OD, right eye; OS, left eye.

**Figure S4.** Mean frequency of artificial tear use (A) and the percentage of patients using artificial tears (B).

Data for frequency of artificial tear use are presented as mean ± standard deviation.

LCD, lutein, zeaxanthin isomer, curcumin, and vitamin D3.

**Table S1.** Inclusion and exclusion criteria.

| **Inclusion criteria** | **Exclusion criteria** |
| --- | --- |
| 1. Male and/or female subjects aged 18–65 years (inclusive) 2. Clinical diagnosis of symptomatic DED at baseline confirmed by a licensed optometrist based on the presence of irritation, foreign body (sandy) sensation, feeling of dryness, itching, non-specific ocular discomfort, and altered/effected QoL at least in any one of the eyes 3. In good health and free from any clinically significant disease, other than DED, that might interfere with the study evaluations 4. Met the following criteria in at least one eye, as determined by a licensed optometrist: (1) Schirmer’s test without anesthesia ≤10 mm and (2) symptomatic DED based on OSDI test score of 12–40 5. Any of the following in at least one eye, as determined by a licensed optometrist: (1) TBUT ≤10 seconds, (2) tear osmolarity ≥316 milliosmole/L, and (3) fluorescein corneal staining ≥1 and <3 6. Willing to provide written consent 7. Willing and able to understand and comply with the requirements of the study, administer the study product as instructed, return for the required treatment period visits, comply with therapy prohibitions, and be able to complete the study | 1. Pregnant, nursing, or planning a pregnancy within the study participation period 2. History of allergy or sensitivity to lutein, zeaxanthin, curcumin or Vitamin D3, related compounds or any component of the formulation 3. Presence of severe DED, with complications such as perforated corneal ulcer, uveitis, or glaucoma, that, in the investigator's opinion, may interfere with DED evaluation 4. Current evidence of ocular infections or inflammatory conditions, such as acute conjunctivitis, or other medical conditions that, in the investigator’s opinion, would cause unnecessary risk by participation and/or compromise the integrity of study data 5. Poorly controlled diabetes mellitus, rheumatoid arthritis, and systemic lupus erythematosus 6. Herpetic eye disease 7. Chronic infection of the lacrimal gland 8. Laser in situ keratomileusis 9. Poorly controlled hypertension (>140/96 mmHg) 10. Evidence of malignancy 11. Major systemic illness necessitating long-term drug treatment, including psychological disorders 12. Concurrent serious hepatic dysfunction or renal dysfunction, uncontrolled pulmonary dysfunction (asthma, COPD, etc.), or other concurrent severe disease 13. Any ocular trauma or surgery that may affect corneal sensitivity and/or normal tear distribution (e.g., cataract surgery, refractive surgery) within the 6 previous months to study inclusion 14. Inability to swallow soft gel capsules 15. Use of lutein, zeaxanthin, curcumin, or vitamin D3 on prescription for other medical indications or for health-conscious reasons 16. Use of steroids or hormone replacement therapy, or any other medication that may adversely affect the outcome of the study 17. Current use of aspirin or anticoagulant therapy 18. Any other medical condition that might adversely impact patient safety or confound study results |

COPD, chronic obstructive pulmonary disease; DED, dry eye disease; OSDI, Ocular Surface Disease Index; QoL, quality of life; TBUT, tear film break-up time.

**Table S2.** Investigational Product Specifications.

| **Product** | **LCD*** | **Placebo** | |
| --- | --- | --- | --- |
| **Dosage Form** | Soft gel capsule (approximate weight 670 mg) | Soft gel capsule (approximate weight 670 mg) | |
| **Composition** | - Capsule weight approximately  670 mg - 40 mg of micronized marigold concentrate^†^ providing 20 mg lutein and 4 mg zeaxanthin - 238 mg micronized curcumin extract 95% providing 200 mg curcuminoids - 1.5 mg of Vitamin D3 providing 600 IU - Approximately 386 mg of excipients | - Approximately 670 mg soybean oil |  |
| **Test Ingredient** | - 20 mg lutein - 4 mg zeaxanthin - 200 mg curcuminoids - 600 IU vitamin D3 | - None | |
| **Route** | Oral | Oral | |
| **Dosing Regimen** | QD | QD | |
| **Treatment duration** | 56 days | 56 days | |
| **Manufacturer** | OmniActive Health Technologies Ltd | OmniActive Health Technologies Ltd | |

*Production of the LCD supplement incorporated novel formulation techniques, including micronization of the active herbal ingredients by jet milling to reduce particle size, and a combination of functional oils to support improved absorption in the small intestine (Muz at al. *Pharmaceuticals (Basel)* (2020) 13(10):295). ^†^Optimized marigold extract as a source of lutein and zeaxanthin (Muz at al. *Pharmaceuticals (Basel)* (2020) 13(10):295).

at a 5:1 ratio as occur in fruits and vegetables.

LCD, lutein, zeaxanthin isomer, curcumin, and vitamin D3; QD, once-daily

**Table S3.** Detailed assessments for in Ocular Surface Disease Index, tear film break-up time and Standard Patient Evaluation of Eye Dryness

| **Assessment** | **Procedures used** |
| --- | --- |
| OSDI | - The OSDI questionnaire consisted of 12 questions across three subscale domains measuring:   1. Frequency of ocular symptoms (sensitivity to light, grittiness, sore/painful eyes, blurred vision, and poor vision; questions 1–5)   2. Vision issues impacting daily activities (reading, television viewing, computer work, and night-time driving; questions 6–9)   3. Ocular discomfort triggered by environmental factors (wind, low humidity, and air conditioning; questions 10–12) - Participants used a linear scale of 0 (none of the time), 1 (some of the time), 2 (half of the time), 3 (most of the time), or 4 (all the time) when responding, to assign a score to each question - OSDI score was calculated as the sum of scores multiplied by 25 and divided by the number of questions answered, with this method applied to each of the three domain subscales for domain scores and for the entire questionnaire for total score - Participants could respond not applicable (N/A) to questions for subscales 2 and 3 (questions 6-12) and these responses were not counted as an answered question |
| TBUT | - The established TBUT assessment was used to assess tear break-up time, rather the newer NITBUT, based on familiarity of the four centres included in the study as part of their routine practice and to minimise inter-centre variability caused by implementing a less familiar test - Participants sat at a slit lamp looking straight ahead with their chin resting on a chin pad - One drop of Purilens saline solution was applied to the stain-impregnated end of a sterile fluorescein paper strip and applied to the inside of the lower eyelid while the participant looked upward. Fluorescein volume was not controlled using a pipette or similar measure - The participant was instructed to blink multiple times to distribute the fluorescein over the ocular surface and the eyes were scanned after 15 seconds - Participants were instructed not blink as their eyes were examined under a cobalt blue light with a yellow filter by a slit lamp in a dim room - For each test, the time from the last blink to the first appearance of a distinct break-up in tear film was recorded in seconds for both eyes |
| SPEED | - The SPEED questionnaire consisted of 8 items measuring the severity of the DED symptoms: burning, dryness, eye fatigue, grittiness, irritation, scratchiness, soreness, and watering - Participants reported symptom frequency using an analog scale of 0 (never), 1 (sometimes), 2 (often), and 3 (constant) - Participants reported symptom severity using an analog scale of 0 (no problem), 1 (tolerable), 2 (uncomfortable), 3 (bothersome), and 4 (intolerable) to - SPEED scores were calculated by summing the frequency and severity scores, with a total range of 0–28 - The sum of responses for the frequency and severity domains of the questionnaire gave specific scores for symptom frequency (maximum of 12) and severity (maximum of 16) for each participant |
| Corneal and conjunctival staining | - Both eyes were examined for corneal and conjunctival staining following the application of fluorescein for TBUT assessment: One drop of Purilens saline solution was applied to the stain-impregnated end of a sterile fluorescein paper strip and applied to the inside of the lower eyelid while the participant looked upward. Fluorescein volume was not controlled using a pipette or similar measure - Severity of ocular surface damage for the cornea and conjunctiva was assessed using the Efron scale based on coloration of the staining using an analogue scale: 0 (no staining), 1 (mild or superficial stippling), 2 (moderate or punctate staining), 3 (severe abrasion or erosion) |

NITBUT, non-invasive tear break-up time; OSDI, Ocular Surface Disease Index; SPEED, Standard Patient Evaluation of Eye Dryness; TBUT, tear film break-up time.

**Table S4.** Mean (SD) frequency of artificial tear use.

| **Days** | **LCD**  **(Mean ± SD)** | **Placebo**  **(Mean ± SD)** |
| --- | --- | --- |
| **Day 0** | 2.40 ± 2.13 | 2.54 ± 1.72 |
| **Day 14** | 2.56 ± 1.74 | 2.45 ± 1.96 |
| **Day 28** | 2.21 ± 1.71 | 2.48 ± 1.99 |
| **Day 56** | 2.29 ± 1.68 | 2.11 ± 1.45 |

LCD, lutein, zeaxanthin isomer, curcumin, and vitamin D3; SD, standard deviation.

**Table S5.** Standard error of the mean values for outcomes assessed.

| **Outcome** | **Days** | **LCD**  **SEM*** | **Placebo**  **SEM*** |
| --- | --- | --- | --- |
| **Schirmer’s test (mm) OD** | **Day 14 Day 28 Day 56** | 0.34 0.29 0.37 | 0.17 0.15 0.15 |
| **Schirmer’s test (mm) OS** | **Day 14 Day 28 Day 56** | 0.26 0.30 0.34 | 0.15 0.15 0.16 |
| **Schirmer’s test (mm) both** | **Day 14 Day 28 Day 56** | 0.25 0.19 0.30 | .13 0.13 0.13 |
| **TBUT (s) OD** | **Day 14 Day 28 Day 56** | 0.21 0.23 0.25 | 0.13 0.12 0.19 |
| **TBUT (s) OS** | **Day 14 Day 28 Day 56** | 0.16 0.17 0.22 | 0.13 0.13 0.15 |
| **TBUT (s) Mean of both** | **Day 14 Day 28 Day 56** | 0.15 0.15 0.19 | 0.11 0.10 0.15 |
| **OSDI total score (au)** | **Day 14 Day 28 Day 56** | 1.32 1.57 1.33 | 1.41 1.27 1.39 |
| **SPEED total score (au)** | **Day 14 Day 28 Day 56** | 0.49 0.52 0.50 | 0.41 0.50 0.48 |
| **Corneal staining (au) OD** | **Day 56** | 0.05 | 0.06 |
| **Corneal staining (au) OS** | **Day 56** | 0.05 | 0.08 |
| **Corneal staining (au) Mean of both** | **Day 56** | 0.05 | 0.05 |
| **Conjunctival staining (au) OD** | **Day 56** | 0.07 | 0.06 |
| **Conjunctival staining (au) OS** | **Day 56** | 0.07 | 0.06 |
| **Conjunctival staining (au) Mean of both** | **Day 56** | 0.06 | 0.06 |
| **Tear Osmolarity (mOsms/L) OD** | **Day 56** | 0.09 | 0.6 |
| **Tear Osmolarity (mOsms/L) OS** | **Day 56** | 1.04 | 0.53 |
| **Tear Osmolarity (mOsms/L) Mean of both** | **Day 56** | 0.83 | 0.48 |

SEM is the difference between means of the given time point and Day 0.

au, arbitrary units; OSDI, ocular severity disease index; LCD, lutein, zeaxanthin isomer, curcumin, and vitamin D3; SEM, standard error of the mean; SPEED, Standard Patient Evaluation of Eye Dryness; TBUT, tear break-up time.
